# Health Care Applicability of a Patient-Centric Web Portal for Patients’ Medication Experience

**DOI:** 10.2196/jmir.5813

**Published:** 2016-07-22

**Authors:** Song Hee Hong, Woojung Lee, Yazed AlRuthia

**Affiliations:** ^1^ College of Pharmacy Seoul National University Seoul Republic Of Korea; ^2^ College of Pharmacy King Saud University Riyadh Saudi Arabia

**Keywords:** patient-physician communication, medication experience outcomes, patient reports, Internet, patient-centered practice, Web portal

## Abstract

**Background:**

With the advent of the patient-centered care paradigm, it is important to examine what patients’ reports of medication experience (PROME) mean to patient care. PROME available through a Web portal provide information on medication treatment options and outcomes from the patient’s perspective. Patients who find certain PROME compelling are likely to mention them at their physician visit, triggering a discussion between the patient and the physician. However, no studies have examined PROME’s potential applicability to patient care.

**Objective:**

This study aimed to examine older (≥50 years) adults’ perceptions of the health care applicability of a hypothetical PROME Web portal. Specifically, this study investigated whether PROME would facilitate patient-physician communication, and identified the preferred reporting items and the trusted sponsors of such a PROME Web portal.

**Methods:**

We used a cross-sectional, self-administered, 5-point Likert scale survey to examine participants’ perceptions of a hypothetical PROME Web portal that compared PROME for 5 common antihypertensive medications. Between August and December 2013, we recruited 300 members of 7 seniors’ centers in a metropolitan area of a southeastern state of the United States to participate in the survey.

**Results:**

An overwhelming majority of study participants (243/300, 81.0%) had a favorable perception of PROME’s health care applicability. They were mostly positive that PROME would facilitate patient-physician communication, except for the perception that physicians would be upset by the mention of PROME (n=133, 44.3%). Further, 85.7% (n=257) of participants considered the PROME information trustworthy, and 72.0% (n=216) were willing to participate by reporting their own medication experiences. Study participants wanted the PROME Web portal to report the number of reviews, star ratings, and individual comments concerning different medication attributes such as side effects (224/809, 27.7%), cost (168/809, 20.8%), and effectiveness (153/809, 18.9%). Finally, the PROME Web portal sponsorship was important to participants, with the most trusted sponsor being academic institutions (120/400, 30.0%).

**Conclusions:**

PROME, if well compiled through Web portals, have the potential to facilitate patient-physician communication.

## Introduction

There is now consensus that patients’ reports of their health experience reflect quality of care [1]. Accordingly, payers such as the Centers for Medicare and Medicaid Services in the United States and the National Health Service in the United Kingdom use patient-reported experience measures for the purpose of performance evaluation and compensation of health care providers [1-4]. However, no studies have examined what patients’ reported medication experience means to patient care. The frequent use of medication indicates that such information could have great potential to affect patient care, especially in the management of chronic diseases.

Patients’ reports of medication experience (PROME) are likely to facilitate patient-physician communication. Patients who find certain PROME compelling are likely to mention them at their physician visit. The mention then would trigger a discussion between the patient and the physician, just as direct-to-consumer advertising (DTCA) does. One-third of the participants in a US Food and Drug Administration (FDA) survey of DTCA said that they initiated conversations with their physicians because of advertising [5]. PROME may more effectively trigger patient-physician communication than DTCA because PROME comes from users, while advertising comes from sellers. Increased patient-physician communication is key to advancing patient-centered practice.

The potential for PROME to influence patient care has given birth to several Web portals such as AskaPatient [6] and DrugRatingz [7]. WebMD [8] and ConsumerReports [9] have also begun to compile patient reviews of medications, along with drug information. The Web portals provide a venue for patients to report their medication experience in terms of effectiveness, side effects, and costs. Moreover, PROME Web portals can present information according to medication classes and patient characteristics. Patients who are browsing those PROME Web portals can easily learn about what medication options have received favorable ratings from which group of patients. When patients come across a report from other patients in the same situation, they are likely to act on the information included in the report [10]. These medicine-focused social media, with a large volume of high-quality first-hand patients’ reviews, are also considered to be a promising data source for understanding patients’ medication experience [11].

With growth in the number of PROME Web portals comes a critical need to examine PROME’s potential applicability to health care. In this study, we aimed to determine participants’ perceptions of whether PROME would facilitate patient-physician communication, and to identify the preferred patient-reporting items and trusted sponsors of such a PROME Web portal.

## Methods

### Study Design and Participants

We used a cross-sectional survey to examine participants’ perceptions of PROME. A detailed description of the survey procedure is given in a doctoral dissertation [12].We scheduled visits to conduct the survey with the coordinators of 7 seniors’ centers in a metropolitan area of a southeastern state in the United States. On each visit, 2 research assistants recruited study participants into a reserved private room and explained the purpose of the study, along with the rights of the participants. Those who completed the survey received a US $20 grocery gift card as an appreciation for their participation. Data collection was started in August 2013 and was continued until we reached our goal of 300 completed surveys in December 2013. Before beginning the study, the University of Tennessee Health Science Center Institutional Review Board determined the study to be exempt from their oversight.

### Survey Instrument

**Figure 1:**
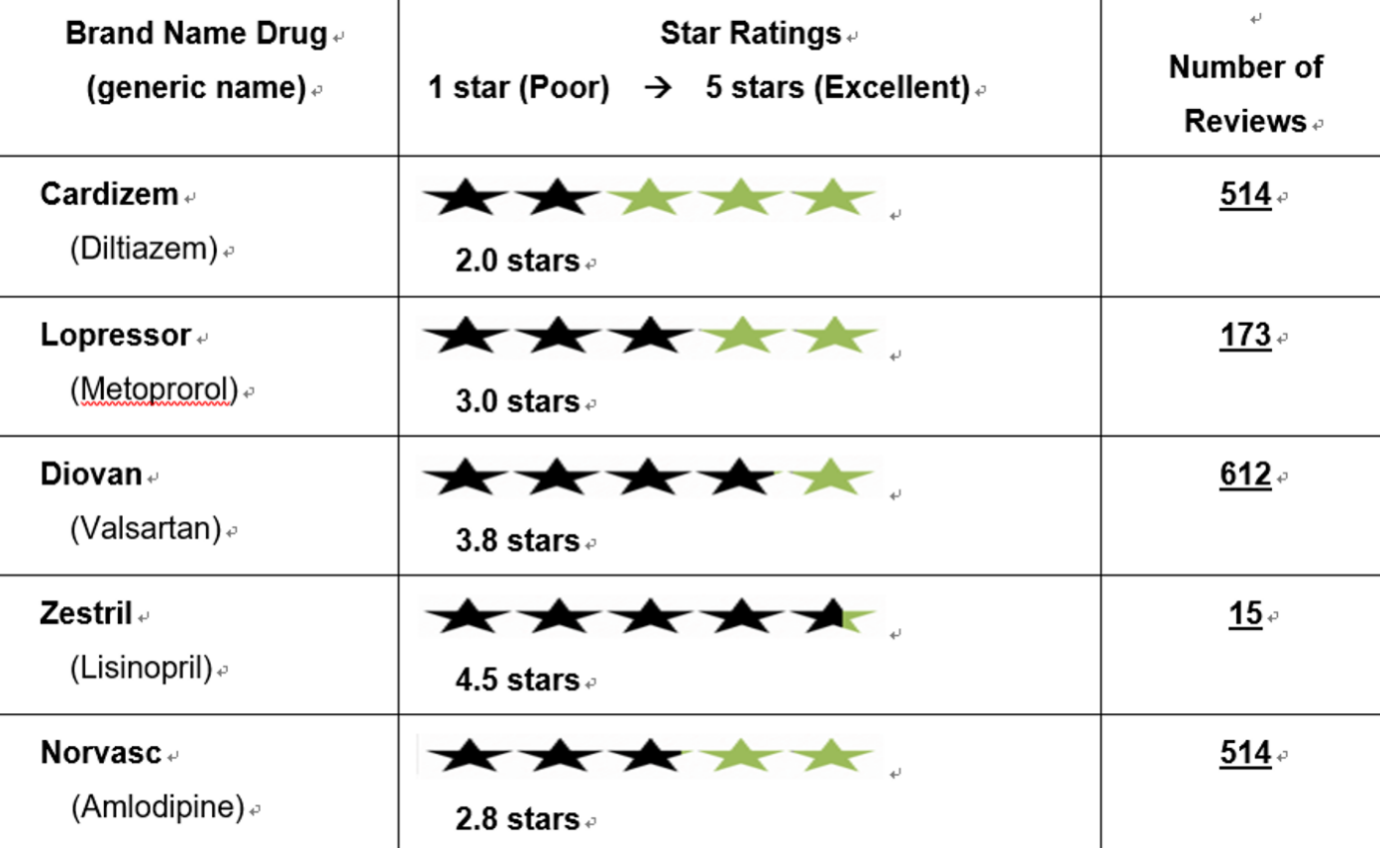
Hypothetical summary of patients' reports of medication experience (PROME) with antihypertensive medications presented to survey participants.

The survey instrument used for this study contained a chart from a hypothetical PROME Web portal that compared PROME for 5 common antihypertensive medications (Figure 1). The chart used arbitrary 5-star ratings and included the number of people who supposedly gave reviews for each medication. We asked for the following sociodemographic information from the participants: sex, age, years of education, race, family member(s) they live with, and income. We asked participants 17 questions in total: 6 demographic questions and 11 questions about the PROME Web portal.

Referring to the PROME chart, we surveyed participants’ perceptions of its potential applicability to health care using 7 questions: (1) 1 question on overall usefulness, (2) 4 questions on patient-physician communication, and (3) 2 final questions: 1 on the perceived credibility of the information provided by the PROME Web portal, and 1 on the willingness of the survey participants to provide their own medication experiences to a PROME Web portal. The 4 questions on patient-physician communication concerned the likelihood for patients to mention PROME to their physician, the likelihood for PROME to facilitate the communication, the perceived likelihood for physicians to be upset by the PROME mention, and the likelihood for patients to ask their physicians to prescribe the PROME-recommended medication. All the questions were answered on a 5-point Likert scale with the following choices: definitely, very probably, probably, probably not, and definitely not. Previous surveys on the effect of DTCA on patient-physician communication indicated that there would be more positive than negative evaluations [5]. We used the unbalanced scale to provide more discrimination between positive evaluations [13]

The survey also had 2 questions on the preferred reporting items, that is, what information the participants wanted to see in the PROME Web portal. The first question asked participants to indicate which reporting items (star ratings, number of reviews, and individual comments) they believed valuable. The second question asked participants to indicate any medication attributes (effectiveness, side effect, food interaction, convenience, and cost) they believed the PROME Web portal should report.

Lastly, the survey had 2 additional questions on PROME Web portal sponsorship, that is, what entity study participants believed should sponsor the PROME Web portal. The first question asked participants to rate the importance of PROME Web portal sponsorship on a 5-point Likert scale. The second question asked participants to indicate any types of sponsors (academic institutions, nonprofit foundations, chain pharmacies, health information companies, and drug plans) they would trust.

### Statistical Analysis

For the participants’ perceptions of PROME’s potential applicability to health care, we reported the percentages selected for each of the 5 response choices as a bar graph. Further, we dichotomized the 5 response choices into having a positive (definitely, very probably, and probably) or a negative perception (probably not or definitely not), and then estimated the probability of having a positive perception, along with 95% CIs. We constructed pie charts to describe the frequency distribution of medication attributes that study participants picked as important elements of PROME coverage and to generate the frequency distribution of PROME Web portal sponsors that study participants picked as trusted. We used chi-square tests to determine whether participants’ perceptions varied with their sociodemographic characteristics at a significance level of 5%. Analyses were conducted using SAS software, version 9.3 (SAS Institute Inc).

## Results

### Study Participants

We contacted older adults (≥50 years) who were members of seniors’ centers in a metropolitan area in a southeastern US state to participate in our survey. Table 1 lists the characteristics of the 300 older adults who completed our survey. They had a mean age of 71.95 years (SD 8.65), were mostly non-Hispanic white (164/300, 54.7%) and female (231/299, 77.3%), with at least some high school education (287/297, 96.6%) and annual incomes of at least US $10,000 (239/262, 92.3%).

**Table 1:** Sociodemographic characteristics of the study participants (N=300).

Characteristics		No. (%)^a^
**Age range (years) (n=295)**		
	50–59	9 (3.1)
	60–69	118 (40.0)
	70–79	105 (35.6)
	80–89	56 (19.0)
	≥90	7 (2.4)
**Sex (n=299)**		
	Male	68 (22.7)
	Female	231 (77.3)
**Education (n=297)**		
	Middle school or less	10 (3.4)
	High school or graduate	123 (41.4)
	Some college	92 (31.0)
	College graduate or higher	72 (24.2)
**Race (n=300)**		
	Non-Hispanic white	164 (54.7)
	Non-Hispanic black	121 (40.3)
	Other^b^	15 (5.0)
**Living status (n=295)**		
	Alone	132 (44.7)
	With daughter or son	31 (10.5)
	With companion or sibling	21 (7.1)
	With spouse	99 (33.6)
	Other^c^	12 (4.1)
**Income (US$) (n=262)**		
	<10,000	23 (7.7)
	10,000–29,000	111 (37.0)
	30,000–49,000	56 (18.7)
	50,000–69,000	41 (13.7)
	≥70,000	31 (10.3)

^a^Some numbers do not add up to 300 because not all participants answered each question.

^b^Other includes Asian, Native American, and Alaskan native.

^c^Other includes living with a parent, a grandson, a niece, or a pet, and living in a retirement community.

### Health Care Applicability of PROME

**Figure 2:**
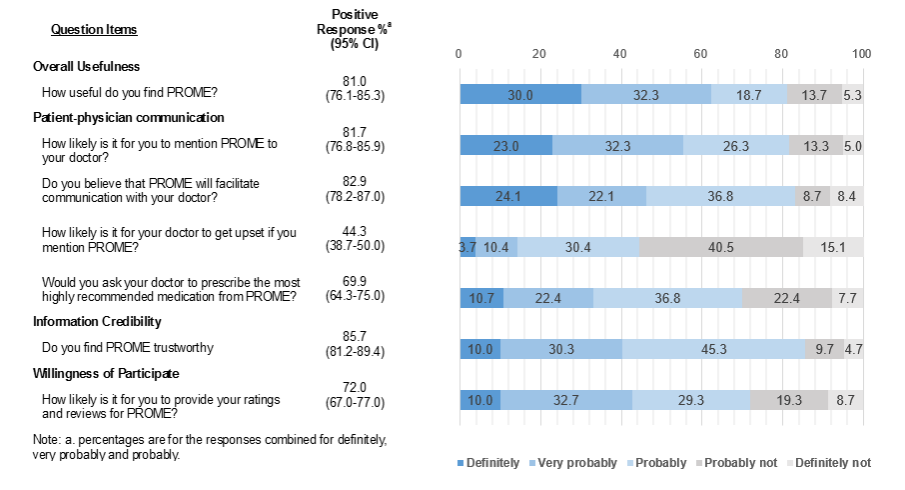
Participants' perceptions of the potential health care applicability of patients’ reports of medication experience (PROME).

As Figure 2 shows, an overwhelming majority of study participants (n=243, 81.0%) were positive about the overall usefulness of PROME; however, the percentage positive decreased to 62.3% (187/300) when excluding probably as the response. As for patient-physician communication, 245 of the 300 participants (81.7%) said that they would mention PROME to their physician (166/300, 55.3% for the responses definitely and very probably), and 248 participants (82.9%) said that PROME would facilitate patient-physician communication (138/299, 46.2% for the responses definitely and very probably). However, 133 participants (44.3%) said that their physician would get upset if they mentioned PROME (42/299, 14.1% for the responses definitely and very probably). Further, 209 (69.9%) of study participants were positive that they would ask their physician to prescribe a PROME-recommended medication. When we further broke down the positive responses to the question on whether physicians would be upset by such a request into probably, very probably, and definitely, we found that more participants chose probably (n=110, 36.8%) than very probably and definitely combined (n=99, 33.1%). In other words, study participants had some reservations about asking their physician to prescribe a PROME-recommended medication.

As for information credibility, most of our study participants (n=257, 85.7%) were positive that PROME information is trustworthy. Further, two-thirds (n=216, 72.0%) were willing to participate in PROME Web portals by providing their own medication experiences. However, for information credibility, more participants responded probably (n=136, 45.3%) than very probably and definitely combined (n=121, 40.3%) (Figure 2).

### Preferred Reporting Items and Sponsorship of the PROME Web Portal

**Figure 3:**
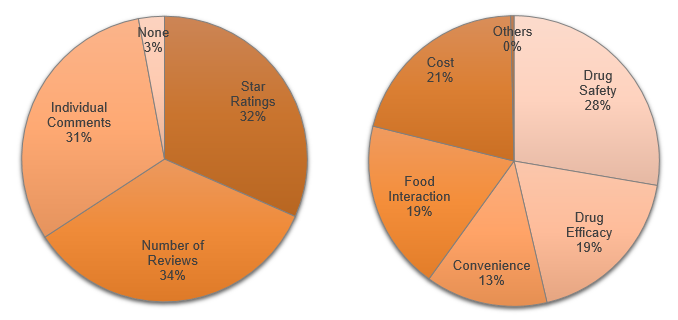
Left: Reporting items that participants believe are valuable (308 responses). Right: Medication attributes on which participants believe patients’ reports of medication experience should report (809 responses).

**Figure 4:**
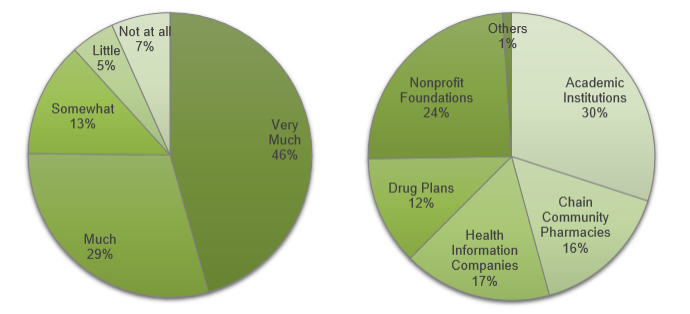
Left: The importance of sponsorship of a Web portal featuring patients’ reports of medication experience (298 responses). Right: The sponsors that participants trust the most (400 responses).

When asked about how PROME should be displayed in a Web portal, of the 308 answers given, participants most frequently picked the number of reviews (n=105, 34.1%), followed by star ratings (n=97, 31.5%) and individual comments (n=96, 31.2%) (Figure 3). However, the difference was negligible. Study participants were also asked to indicate medication attributes (such as effectiveness, side effects, ease of use, costs, and interaction with food) that PROME should cover. Study participants gave 809 answers and picked side effects most often (n=224, 27.7%) and ease of use least often (n=110, 13%). Cost (n=168, 20.8%), effectiveness (n=153, 18.9%), and interaction with food (n=151, 18.7%) were picked almost equally (Figure 3). When asked to indicate the importance of the PROME Web portal’s sponsorship, 263 of 298 participants (88.2%) said that sponsorship is important (Figure 4). Academic institutions such as the University of Tennessee were viewed as the most trusted sponsors (120/400, 30.0%), followed by nonprofit foundations such as the American Heart Association (97/400, 24.3%) (Figure 4).

### Views on PROME According to Demographic Factors

**Figure 5:**
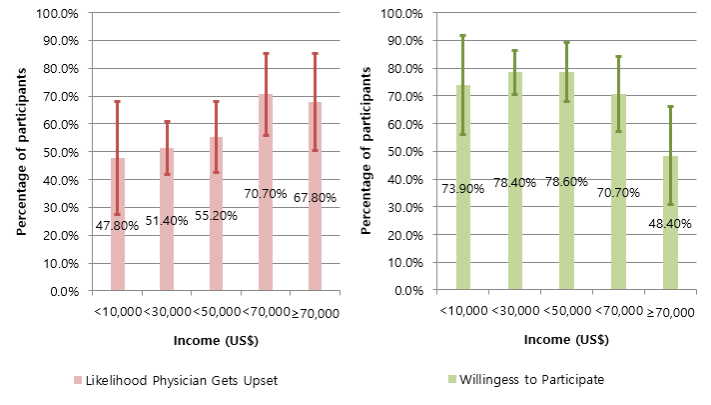
Income effects on participants’ perceptions of the likelihood that physicians would get upset if they mentioned patients’ reports of medication experience (PROME) and their willingness to participate in a PROME Web portal. Error bars indicate 95% CI.

**Figure 6:**
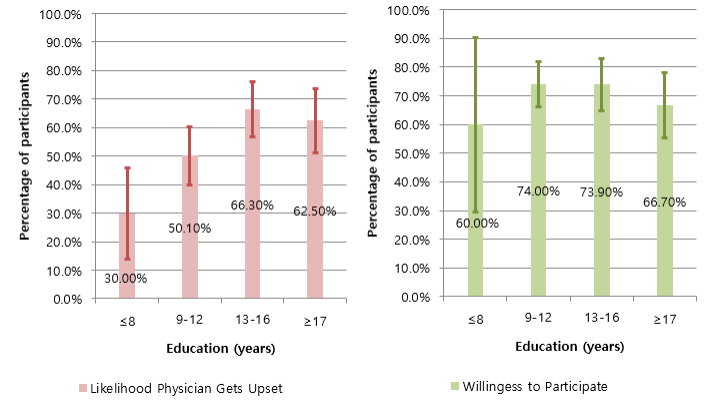
Education effects on participants’ perceptions of the likelihood that physicians would get upset if they mentioned patients’ reports of medication experience (PROME) and their willingness to participate in a PROME Web portal. Error bars indicate 95% CI.

Across all sociodemographic characteristics, study participants were positive that PROME provide useful and credible information to facilitate patient-physician communication (Table 2). Further, their willingness to participate in PROME Web portals remained high across all sociodemographics except for income. Study participants in the highest income bracket (over US $70,000 per year) were least willing to participate in PROME Web portals (*P*=.02) (Figure 5). However, the perceived likelihood that physicians would be upset was significantly different across several demographic variables. With increased education, study participants were more likely to believe that physicians would get upset by a mention of PROME (3/10, 30% for people with middle school education or less and 61/92, 66% for people with some college) (Figure 6). This trend was also present with income levels: the higher the income, the more likely the participant was to believe physicians would get upset by a mention of PROME (Figure 5). Likewise, non-Hispanic whites were more likely than non-Hispanic blacks to believe that physicians would get upset by a mention of PROME.

**Table 2:** Percentage of positive views^a^ on the potential applicability of a Web portal featuring patients’ reports of medication experience (PROME) to health care, by demographic characteristic (N=300).

Characteristics		No.	Items relating to perceptions of PROME Web portal applicability, % (95% CI)				
			Overall usefulness	Patient-physician communication^b^	Physician getting upset	Information credibility	Willingness to participate
			n (%) 95% CI	n (%) 95% CI	n (%) 95% CI	n (%) 95% CI	n (%) 95% CI
**Sex**							
	Female	231	187 (81.0%) 75.9–86.0	168 (72.7%) 66.5–78.4	134 (58.0%) 51.6–64.4	197 (85.3%) 80.7–90.0	162 (70.1%) 64.2–76.1
	Male	68	55 (80.9%) 71.5–90.2	53 (77.9%) 66.2–87.1	33 (48.5%) 36.7–60.4	59 (86.8%) 78.5–95.0	53 (78.0%) 67.8–88.1
**Education (years)**							
	≤8	10	9 (90.0%) 78.0–100.0	8 (80.0%) 70.1–91.9	3 (30.0%) 13.1–45.0	7 (70.0%) 41.6–98.4	6 (60.0%) 29.6–90.4
	9–12	123	102 (83.3%) 76.1–90.6	89 (72.6%) 63.9–81.2	62 (50.1%) 40.9–61.0	107 (87.0%) 81.1–93.0	91 (74.0%) 66.2–81.7
	13–16	92	69 (75.0%) 66.2–83.9	64 (70.1%) 61.4–80.0	61 (66.3%) 56.6–76.0	80 (87.0%) 78.3–93.1	68 (73.9%) 64.9–82.9
	≥17	72	58 (80.6%) 71.4–89.7	55 (76.4%) 66.6–86.2	45 (62.5%) 51.3–73.7	61 (84.7%) 76.4–93.0	48 (66.7%) 54.6–77.3
**Race**							
	Non-Hispanic white	164	133 (81.1%) 75.1–87.1	117 (71.3%) 64.4–78.3	103 (62.8%) 55.4–70.2	139 (84.8%) 78.3–89.9	110 (67.1%) 59.9–74.3
	Non-Hispanic black	121	97 (80.0%) 72.8–87.2	93 (76.7%) 69.1–84.2	60 (49.2%) 40.2–58.1	103 (85.1%) 78.9–91.5	94 (77.7%) 70.3–85.1
	Other^c^	15	13 (87.5%) 71.3–100.0	12 (81.3%) 54.4–96.0	5 (33.3%) 9.5–54.0	15 (100.0%) 100.0–100.0	12 (80.0%) 59.8–100.0
**Income (US$)**							
	<10,000	23	17 (73.9%) 56.0–91.7	18 (78.3%) 61.4–95.1	11 (47.8%) 27.4–68.2	16 (69.6%) 50.8–88.4	17 (73.9%) 56.0–91.9
	10,000–29,000	111	94 (84.4%) 77.6–91.2	92 (82.6%) 75.5–89.7	57 (51.4%) 42.0–60.8	99 (89.2%) 83.4–95.0	87 (78.4%) 70.7–86.0
	30,000–49,000	56	47 (84.5%) 75.2–93.8	41 (74.1%) 62.9–85.4	31 (55.2%) 42.4–68.0	50 (87.5%) 78.8–96.2	44 (78.6%) 67.8–89.3
	50,000–69,000	41	33 (80.5%) 68.4–92.6	28 (68.3%) 54.1–82.5	29 (70.7%) 54.5–83.9	35 (85.4%) 74.6–96.2	29 (70.7%) 56.8–83.9
	70,000	31	22 (71.0%) 55.0–87.0	18 (58.1%) 40.7–75.4	21 (67.8%) 48.6–83.3	25 (80.7%) 62.5–92.6	15 (48.4%) 30.8–66.0

^a^The percentage of positive answers is for the response choices of probably, very probably, and definitely combined.

^b^The 3 items related to patient-physician communication were combined because individually they did not have any significant demographic variations. The percentages of positive responses were for those with the combined score 9, the score when all 3 items were given a rating of 3 (a response of probably on the 5-point scale).

^c^Other includes Asian, Native American, and Alaskan native.

## Discussion

PROME would greatly affect patient care if patients consider the PROME information to be useful and credible, and are willing to contribute their own reviews and ratings for PROME. This study found that an overwhelming majority of study participants (81.0%) were at least positive that PROME are overall useful; 62.3% were definitely or very probably positive. The positive perception implies that older patients are in need of medication information written by the patient, for the patient. Patients who have received treatment under the paternalistic care environment are longing for information about overall treatment processes and alternative treatment options [14].

More than 80% of study participants were positive that PROME would facilitate patient-physician communication by triggering a mention of PROME. This percentage is much higher than those found in FDA-sponsored surveys of patients regarding DTCA. Only one-third of the participants in the FDA survey conducted in 2002 said that advertisements had encouraged them to ask a question of their physician, while 43%, a decline from 62% in 1999, felt that advertisements helped them have better discussions with their doctors [5]. Evidently, PROME have a greater potential to influence patient-physician communication than advertisements. Further, the likelihood of asking for a specific brand was also influenced quite differently by PROME and advertisements. In our study, more than two-thirds of study participants (69.9%) said that they would ask their physician to prescribe a PROME-recommended brand, compared with 39% of the FDA’s survey participants stating that they would ask for an advertisement-recommended brand.

It is interesting that participants perceived that physicians would be upset by their mention of PROME. While our survey did not provide data to explain this perception, perhaps the participants believed that they would be challenging the physician’s authority to prescribe by mentioning PROME-recommended drug therapy options. Alternatively, participants might have had past experience with physicians who were reluctant to discuss potential drug therapy options. However, studies have shown that physicians are in fact willing to discuss therapy options with patients [15,16]. It may be, then, that participants felt that physicians, in general, welcome questions on health issues but may not be as welcoming to those on drug therapy options, especially a request for a specific brand.

Patients often regard a piece of information as trustworthy when it comes from other patients with a similar condition [10]. However, study participants had some reservations concerning the trustworthiness of the PROME information. Traditionally, online information has been viewed as less trustworthy than print information [17]. Further, many are reluctant to trust online information [18,19], especially older adults, such as the study participants, who trust online health information less than do younger adults [20,21].

As for willingness to participate in PROME Web portals, 72.0% of study participants said they were willing to provide their own reviews and ratings. It is remarkable that such a high percentage of seniors were willing to participate in PROME Web portals. It may reflect the ongoing trend of a rapidly increasing senior population searching for health-related information on the Internet [22]. It could also reflect the patient-centric health care paradigm, where patients are actively seeking other patients’ experience. According to a study of PatientsLikeMe, patients refer to other patients’ experience to better understand and control their diseases [10]. Thus, it follows that patients who seek other patients’ experience would be willing to participate in PROME Web portals.

Patient reports, just like any other consumer review, can be summarized in 3 reporting items (star ratings, number of reviews, and individual comments). Study participants indicated that the PROME Web portal should provide information on all 3 reporting items. The 3 items evidently capture different aspects of patients’ medication experience. Without capturing all these aspects, PROME may not successfully reflect patients’ true medication experience.

A well-designed PROME Web portal should also provide information on all the attributes of medication experience, such as effectiveness, side effects, ease of use, costs, and interaction with food. Participants picked side effects, followed closely by cost, effectiveness, and interaction with food, as the most important attributes to report in a PROME Web portal. Ease of use was least often picked. It is not surprising that study participants were most interested in side effects. The FDA’s study also reported that far more people look for information on side effects than on benefits (61% vs 10%) [5]. What is rather surprising is that more people in this study wanted the PROME Web portal to report on drug cost than on effectiveness (20.8% vs 18.9%). This finding is starkly different from the FDA’s survey, where few people (4%) wanted cost information from advertisements [5]. Patients seem to put more trust in PROME than in advertisements for drug cost information. Chronic diseases are prevalent among older adults and require ongoing medication management. Older patients who live on a fixed income could face substantial financial distress due to drug cost [23,24]. Thus, these patients would naturally seek information on drug cost.

Information available on the Internet has a high chance of misleading people [19,25]. Without a reputable sponsor, online information is difficult to trust [26]. As many as 90% of study participants said that it is important who sponsors the PROME Web portal. Evidently, sponsorship makes a big impact on patients’ willingness to trust online medical information. Patients rarely read online information unless there is a transparent and dependable sponsorship [27].Without an address or a phone number of the sponsor, patients simply do not trust online information [28]. As for a trusted sponsor of the PROME Web portal, in this study participants picked academic institutions as the most trusted. Another study also reported that the most trusted sponsor of health information is a university [29]. According to our participants, private organizations such as WebMD, ConsumerReports, and chain pharmacies were viewed as least trusted. People seem to perceive that private organizations act in their own interests ahead of patients’ interests.

Across all demographics, most study participants had a favorable view of the health care applicability of PROME. Meanwhile, about one-fifth of study participants did not agree with the usefulness of PROME. Study participants in the highest income bracket (over US $70,000 per year) showed the lowest willingness to participate in PROME Web portals (*P*<.05). The inverse relationship could imply that participants with the highest income are most satisfied with the medical information they have access to and thus are least likely to feel the need for PROME Web portals for additional information. Participants with higher income may have easier access to various medical information resources. Further, the perceived likelihood that physicians would get upset by their patients mentioning PROME was significantly higher among people with greater education and income. Moreover, this perception was more apparent among non-Hispanic whites than among non-Hispanic blacks. It would be interesting to know why participants with more education were more likely to perceive that physicians would get upset by a mention of PROME. Perhaps education trains people to be more skeptical.

### Limitations

Our findings should be interpreted carefully because of three potential study limitations. First, we have measured participants’ perceptions based on convenience sampling in one metropolitan area. Participants’ perceptions of the study sample may well have been different from those of the US population as a whole. However, the characteristics of our study participants were similar to the population who were over 50 years of age and lived in a metropolitan area of a southeastern state.

Second, study participants may not have been willing to disclose some information, especially when the information was sensitive because of privacy concerns. For instance, many of the respondents refused to disclose their estimated annual income even though we had guaranteed the anonymity and confidentiality of their responses. While this limitation is common to all survey studies, the study sample could have been more reluctant to release the sensitive information.

Third, this study used an unbalanced 5-point Likert scale (3 positive and 2 negative responses) to elicit study participants’ perceptions of PROME with regard to patient-physician communication. Although this scale provides more discrimination between positive responses [13,30], some neutral responses could have been forced to positive ones. When this happens, dichotomizing the responses into positive and negative responses could inflate the occurrence of positive evaluations. However, the likelihood is minimal considering survey participants are known to choose a response based on a label rather than the position on the 5-point scale [31,32]

### Conclusion

This study found that older participants across most demographics considered PROME to provide useful and credible information to facilitate patient-physician communication, and thus were willing to participate in PROME Web portals by sharing their own medication experiences. These participants also believed that an academic institution should sponsor PROME Web portals. Overall, this study found that there is a need for developing a trustworthy Web portal to systematically compile PROME for older patients to communicate well with their physicians.

## Abbreviations

DTCA: direct-to-consumer advertising
FDA: US Food and Drug Administration
PROME: patients’ reports of medication experience
